# Exploring self-compassion among men seeking weight loss: a thematic analysis

**DOI:** 10.1080/17482631.2025.2577285

**Published:** 2025-11-08

**Authors:** Natalie M. Papini, Nikole D. Squires, Amber Iola Jones, Leah Mundell, Stephen D. Herrmann, Nanette V. Lopez

**Affiliations:** a Department of Health Sciences, Northern Arizona University, Flagstaff, Arizona, USA; b Pathogen & Microbiome Institute, Dr., Northern Arizona University, Flagstaff, AZ, USA; c Department of Anthropology, Dr., Northern Arizona University, Flagstaff, Arizona, USA; d Internal Medicine Department, University of Kansas Medical Center, Kansas City, Kansas, USA; e Kansas Center for Metabolism and Obesity Research, University of Kansas Medical Center, Kansas City, Kansas, USA

**Keywords:** Self-compassion, men’s health, semi-structured interviews, reflexive thematic analysis

## Abstract

**Introduction:**

Self-compassion (SC) is associated with reduced eating disorder pathology, body dissatisfaction, and weight concerns, but most SC interventions and research samples focus on women. Because men often face unique challenges related to weight stigma and masculine norms, SC may be especially valuable in supporting emotion-regulation in health coaching programs. Little is known about how adult men engaged in weight loss interpret and experience SC, limiting the reach of interventions intended to support sustainable health behavior change. As such, the purpose of this study was to qualitatively explore how adult men seeking weight loss with health coaching make sense of SC during weight loss. Specifically, we examined experiences and meanings of SC, the perceived barriers and facilitators to practicing it, and how body image and weight-related experiences shape their understanding and application of SC.

**Methods:**

Using reflexive thematic analysis, we explored how 11 adult men enrolled in a commercial weight-normative health coaching program made sense of self-compassion in the context of body image, dieting, and masculinity. Semi-structured interviews were conducted, transcribed, and analyzed through an inductive, interpretive process emphasizing information power and meaning-making over saturation. Researcher reflexivity and positionality were integrated throughout the analytic process.

**Results:**

Seven themes and 20 subthemes were developed. Key themes included: (1) SC as a skill to be learned and practiced; (2) concordance between SC and self-image, including gender norms; (3) the interplay between SC and body image; (4) SC in action through mindset and behavior; (5) barriers to SC such as weight stigma, toxic masculinity, and dieting cycles; (6) facilitators to SC including life experience and upbringing; and (7) SC as a mask for disordered eating and exercise behaviors.

**Discussion:**

Findings highlight the need to tailor SC interventions to address masculine norms, weight stigma, and internalized bias.

## Introduction

Self-compassion (SC) is a Buddhist concept rooted in ancient Eastern philosophy that is defined as the recognition that suffering, failure, and inadequacies are part of being human and that the self deserves compassion (Neff, 2003a, 2003b). Individuals with higher SC are motivated to respond to personal suffering with kindness rather than unkind judgments or harsh self-criticism (Neff, 2003a). SC comprises three central tenets: 1) self-kindness (acting in a kind and understanding way toward self), 2) common humanity (the ability to recognize feelings of inadequacy or periods of struggle as part of the shared human experience), and 3) mindfulness (willingness to experience thoughts and feelings accurately without becoming absorbed by them or evading them) (Neff, 2003b).

Previous findings indicate that SC can reduce the impact of negative and stressful life experiences, such as those encountered in weight-based stigmatizing experiences (MacBeth & Gumley, 2012). Furthermore, interventions designed to improve SC have resulted in reduced eating disorder pathology, eating concerns, body image concerns, and weight concerns among participants (Kelly & Carter, 2015; Turk & Waller, 2020). Still, little is known about SC among men seeking weight loss services since much of the literature involves primarily female samples (Biber & Ellis, 2019). People in larger bodies who seek weight loss services often have reduced mental health outcomes, increased binge eating, greater body dissatisfaction, higher rates of eating in response to negative emotions, and more distress than people who had not sought treatment and normal-weight controls (Fitzgibbon et al., 1993; Gruszka et al., 2021). As such, it is critical to examine how this sample perceives and recognizes self-compassion in the context of body image, weight, and health behaviors.

Early research on the relationship between SC and weight loss suggests SC can be a facilitator of weight loss, nutrition behaviors, and body image, though the number of studies is limited, and many have methodological limitations (Rahimi-Ardabili et al., 2017). SC was significantly associated with adverse emotional reactions to dietary lapses, so individuals could reduce self-criticism when faced with perceived dietary restriction failures (Thø et al., 2021). Thus, SC may be a viable skill to develop among dieters who experience shame and guilt resulting from lapses and setbacks in their attempts to lose weight. Experimental and review evidence suggests SC could support weight regulation by reducing negative cognitions, improving eating behaviors, and enhancing the effectiveness of mindfulness-based interventions (Brenton-Peters et al., 2021; Egan & Mantzios, 2018; Mantzios & Wilson, 2014; Mantzios & Wilson, 2014). However, no evidence was found to suggest that adding SC to WW™, a digital behavioral weight management program, resulted in significant weight loss among adults (Brenton-Peters et al., 2023). Adults who completed the SC and WW™ program reported increased self-kindness and lowered self-judgment (Brenton-Peters et al., 2023). Still, the nascent evidence of SC in weight loss programs suggests that the mechanism by which SC results in weight change serves as an emotion regulation strategy to mitigate eating pathology and poor body image, which can also improve dietary behaviors (Rahimi-Ardabili et al., 2017; Turk & Waller, 2020).

Despite the dangers and pitfalls of dieting, such as weight regain (~70−90% of people regain weight lost) (Hall & Kahan, 2018; Kraschnewski et al., 2010; Wing & Phelan, 2005), increased eating disorder risk (Tylka et al., 2014), and directly contributing to weight stigma (Steinberg & Bohon, 2022), dietary restriction is still common among adults in the United States (Stierman et al., 2020). Qualitative and meta-analytic work indicate that while men tend to report higher SC than women (Yarnell et al., 2015), cultural and gender norms could influence how it is expressed (Egan & Mantzios, 2018; Heath et al., 2017). In a qualitative study examining the lived experiences of individuals with muscle dysmorphia (preoccupation with lack of muscularity and leanness that leads to significant distress), findings showed most participants were persistent dieters who tracked calories, macronutrients and displayed obsessive tendencies regarding protein target ranges (Martenstyn et al., 2022). In particular, men who struggle to regulate eating patterns are more likely to seek help in the form of weight loss treatment rather than seeking treatment for disordered eating (Mars et al., 2019; Westerberg & Waitz, 2013). As such, rather than focusing on how to utilize SC to increase an individual's capacity for weight loss, it may be prudent to examine instead how SC may improve disordered eating behaviors and help people cope with weight stigma or internalized weight stigma, all of which occur in higher rates in adults seeking weight loss services (Giel et al., 2021; Puhl & Himmelstein, 2018; Westerberg & Waitz, 2013).

One limitation of SC research is that most SC interventions target women, especially younger ones, in college or university settings (MacBeth & Gumley, 2012). Most studies that have exclusively incorporated men focus on male university athletes. In one study, SC was applied to male athletes, and significant barriers to implementation for men who endorse traditional masculine norms were observed (Mosewich et al., 2019; Wasylkiw & Clairo, 2018). These findings are aligned with Hegemonic Masculinity Theory (HMT), which posits that the most idealized forms of masculinity are characterized by stoicism, resilience, and denial of weakness (Connell & Messerschmidt, 2005). Reilly and colleagues (2014) observed that shame moderated the negative association between SC and hegemonic masculinity in men, while other work examining SC in men found that men who endorsed traditional masculine norms will have significant barriers to practicing SC and those with more inclusive masculine norms are better able to apply SC in sport (Reilly et al., 2014; Reis et al., 2019).

The Mindful Self-Compassion (MSC) Program (Germer & Neff, 2019) has been widely studied; however, most existent literature includes samples of women. However, one study reported that the MSC program was well-received in a sample of primarily male (71%) racially diverse veterans. This program's participants reported high engagement and completion rates (Serpa et al., 2021). Additionally, the veterans in this program reported significant increases in SC (Serpa et al., 2021). Serpa et al. (2021) findings point to the MSC program's utility as a way to improve their overall SC. However, men may require adaptations to the programming and marketing of such programs (Germer & Neff, 2019). Investigating the patterns of the meaning of SC in men could be one way to tailor existing SC interventions and improve mental well-being in men pursuing weight loss.

While previous work has investigated barriers to SC in women in weight management programs (Jeziorek & Riazi, 2022), no known research qualitatively investigates how adult men seeking weight loss experience SC. Findings from this work could inform future interventions aiming to incorporate SC with male populations. The proposed study aims to qualitatively examine the experience and meaning of SC for males in larger bodies who are actively trying to lose weight through participation in a structured weight loss program, Profile by Sanford (Bell et al., 2021; Papini et al., 2023). The proposed study aims to qualitatively examine the experience and meaning of SC for males in larger bodies who are actively trying to lose weight through participation in a structured weight loss program, Profile by Sanford. Further, we aimed to understand barriers to practicing SC among men and how body image and weight-related experiences shape their understanding and practice of SC.

## Methods

### Participants

This study utilized purposive sampling methods to recruit men seeking weight loss. Participants were eligible to participate in the current study if they had an active membership at Profile by Sanford Health, were 18 years of age or older, identified as male, had a body mass index (BMI) equal to or greater than 25 (BMI of 25−29.9 is categorized as having overweight, BMI of 30 or more is categorized as having obesity), and a history of dieting (defined as at least two previous attempts to control weight). The present study aimed to recruit 8−10 individuals. This sample size is consistent with reflexive thematic analysis studies, which often use small, purposive samples to enable rich, detailed exploration of participants' perspectives (Braun & Clarke, 2021). Sample adequacy in reflexive TA is determined by the depth and complexity of the data concerning the research aims. All participants were Profile by Sanford members, non-Hispanic White men (*n* = 11; 100%), with an average age of 55.72 years (range: 26−77). For additional participant demographics, see Table I.

**Table I.** Participant characteristics (*n* = 11).

Characteristic	Category	*n* (%)
Health coach appointment (≥1/month)		
	Yes	8 (72.72)
	No	3 (27.28)
Membership Duration		
	Less than 6 months	2 (18.18)
	7−11 months	2 (18.18)
	1 + year	7 (63.64)
Loss of Control of Eating		
	Never	1 (9.10)
	<1/week	5 (45.45)
	1 time/week	3 (27.28)
	2−4 times/week	2 (18.18)
	5 + times/week	0 (0.00)

Note: Loss of control of eating was measured using item 8 from the Binge Eating Scale, which assesses the frequency of loss of control of eating (Gormally et al., 1982).

### Recruitment

Purposive sampling methods were used, such that participants were recruited via email through a behavioral weight loss program (Profile by Sanford Health) that incorporates one-on-one health coaching to help individuals set goals around nutrition, physical activity, and lifestyle behaviors. Individuals in the program are encouraged to attend a 30-minute weekly coaching appointment and can meet routinely with a single health coach or different health coaches. Health coaching appointments consist of reviewing past conversations, topics, and goals from the previous week, discussing new education and behavior topics, modifying existing goals, or setting new goals for the next week. Participants were required to complete the survey and interview to be compensated for study participation with a $100 Profile by Sanford Health gift card.

### Procedures

Participants received an email noting the purpose of the study, the time it would take to complete the study, compensation available, and a link to a survey that contained the informed consent, demographic questions (age, ethnicity, income level, length of Profile by Sanford Health coaching membership, current weight, height, number of previous weight loss attempts), and the 12-item self-compassion scale-short form (SCS-SF; Raes et al., 2011). Survey responses were reviewed by study personnel to ensure participant eligibility. Eligible participants were contacted to schedule an interview date and time. Interviews were conducted via Zoom, an innovative videoconferencing platform that is a cost-effective and secure platform for qualitative data collection (Archibald et al., 2019). Participants were informed that the interviewer was affiliated with the health coaching program. The interviewer was not health coaching at the time of this study and had never served as a health coach for any of the participants.

Semi-structured interviews were selected over other methodologies because the ordering of questions is less important, the interviewer has more autonomy to explore interesting topics that arise, and the interviewer can better follow the respondent's interests, concerns, or a piece of their experience. The semi-structured interview process consisted of rapport building, asking interview questions, and implementing member checks. Previous work suggests that video conferencing may facilitate greater connection through participants' comfortability with the setting (where they choose their environment) and mode of communication (Weller, 2017). Member checks, also known as informant feedback or respondent validation, were used to improve the study's accuracy, credibility, and internal validity (Koelsch, 2013).

Before interviewing participants, the principal investigator/interviewer (NP) reviewed the informed consent with participants and asked if they had any questions. Written and verbal informed consent was obtained from all participants. At the time of the interviews, the interviewer (NP) was a doctoral candidate who had completed over 100 hours of qualitative research training before conducting interviews. Further, NP had previously worked as a health coach at the health coaching company and was familiar with the program and general characteristics of the clients served. All interview questions were pilot-tested prior to the start of the study. To review the interview guide, please review Appendix A. Participants were informed that the research aimed to understand their unique experience with SC/their different perspectives of SC and how their unique life experiences may shape their understanding and practice of it. They were assured that there were no “correct” answers to questions, but this was a conversation for the interviewer (NP) to learn more about them and their ideas. Verbal consent was provided after answering any remaining questions about the study. Interview questions were initially based on the work of Jeziorek and Riazi (2022), who used IPA to examine the lived experience of SC in women with overweight who were actively trying to lose weight through commercial weight loss programs. After asking men what they knew about SC and before asking questions about their perspectives of SC, the interviewer provided the following operational definition of SC to all participants: “Self-compassion is acknowledging that suffering, failure, and inadequacies are part of the human condition and that all people—yourself included—are worthy of compassion” (Neff, 2003a, p. 87.). Additional questions were added to incorporate questions about body image and weight stigma experiences specific to the Profile by Sanford Health program. All interview questions were reviewed by an interdisciplinary team, who revised questions for clarity, conciseness, and flow. To see the list of interview questions, see Supplementary Table I.

Interviews were conducted between December 2021- March 2022. All interviews were audio recorded and transcribed verbatim. Interviews lasted between 26 and 87 minutes; the average length was 53.5 minutes. Finally, interviews were conducted in an environment free of disruption that ensured participant confidentiality. This study (#1733353-3) was approved by the Institutional Review Board (IRB) at Northern Arizona University and was determined exempt under 45 CFR 46.101(b). The Consolidated Criteria for Reporting Qualitative Research Reporting guideline (COREQ) was followed (Tong et al., 2007). Study team members with access to data completed personal positionality statements to reflect on and minimize the influence of personal biases that could influence data analysis.

During the preparation of this manuscript, the authors used ChatGPT (OpenAI, GPT−4, March 2025 version) to assist with revising phrasing for clarity and conciseness in the manuscript draft. The tool was not used to generate content or analyze data. All final writing and interpretations were conducted and reviewed by the authors. The use of ChatGPT was limited to editorial support to enhance readability.

### Study design

Participants were adult men (*n* = 11) enrolled in a weight-normative (i.e., focused on weight loss) health coaching program spanning over 150 locations in the United States. Since we sought to explore how participants described and made meaning of their experiences with self-compassion (SC), reflexive thematic analysis was selected as an appropriate and flexible method. Men were recruited using purposive sampling methods, which are commonly used in qualitative research to ensure the inclusion of participants with relevant experiences and perspectives (Braun & Clarke, 2021; Patton, 2015).

### Data analysis

Data were analyzed using reflexive thematic analysis, following Braun and Clarke’s six-phase approach: (1) familiarization with the data, (2) generation of initial codes, (3) construction of candidate themes, (4) review of themes, (5) defining and naming themes, and (6) producing the report (Braun & Clarke, 2006; 2021). The analysis was primarily inductive, allowing codes and themes to be developed from the data without a pre-existing framework. We approached the data from a critical realist perspective, aiming to identify patterned meanings while acknowledging participants’ lived realities.

As reflexive thematic analysis emphasizes the active role of the researcher in meaning-making, we acknowledge our interpretive positioning. The interviewer (NP) is a former health coach at the program from which participants were recruited and identifies as a White woman with training in self-compassion research. This positionality facilitated rapport during interviews and informed the interpretation of themes. To support transparency and reflexive practice, all coders and team members involved in the analysis completed positionality statements before data coding. These reflections were used to promote awareness of personal assumptions and experiences that could influence interpretation. The team also engaged in ongoing reflexive discussions throughout the analysis to ensure a thoughtful, collaborative approach to theme development.

First, two researchers (NS and AJ) independently read and inductively coded 35% of transcripts using Microsoft Excel. Coders noted patterns and meaning units related to self-compassion, body image, and perceptions of health and well-being. The research team (NS, AJ, and NP) met to discuss and synthesize these codes, collaboratively developing an initial coding framework. This framework guided the coding of the remaining transcripts but remained flexible: new codes were added, and existing codes were revised throughout the process. Both coders then applied this framework to the full dataset, engaging in ongoing comparison and discussion to ensure consistency while allowing for new insights to emerge. This iterative process was used to balance rigor and efficiency. Discrepancies were handled through discussion and consensus. The goal of this approach isn’t just discussion, but improved understanding of the data. Adjudication was used when the coders could not come to an agreement. The PI was included in those times to resolve disagreements.

Ongoing team discussions facilitated theme development, in which similar codes were grouped into candidate themes and refined in relation to the full data set. Themes that lacked broader relevance were discarded. Final themes were developed to reflect meaningful patterns across participants' accounts rather than frequency alone. This process represented a form of researcher triangulation, which reduced individual bias and strengthened the credibility of the analysis. The process was recursive and iterative, consistent with reflexive TA's emphasis on the active role of the researcher in theme generation.

The principal investigator (NP) drafted thematic summaries, which were reviewed and confirmed by the broader team. Throughout the process, we followed guidance on maintaining analytic consistency within team-based qualitative designs (Cofie et al., 2022) while remaining reflexive about our positionalities and interpretive contributions.

## Results

Roughly half (*n* = 5) of participants had never heard of SC before the interview. A total of seven themes were developed, including: 1) SC as a Skill, 2) Concordance Between SC and Self-Image, 3) The Interplay of SC and Body Image, 4) SC in Action, 5) Barriers to Practicing SC, 6) Facilitators to Practicing SC, and 7) SC as a Mask. Within these seven themes, 20 subthemes were constructed (See Table II). Various subthemes encapsulated the fundamental essence of every overarching theme, and each subtheme contained illustrative quotes. We prioritized information power (Maltured et al., 2016) and analytic depth over-saturation, which aligns with reflexive TA's emphasis on meaning-making rather than completeness. Because we recruited a highly specific sample for the study aims (adult men seeking weight loss with health coaching) and dialogue quality was strong, information power seemed more appropriate than simply reaching saturation.

**Table II.** Table of themes, subthemes, and definitions.

1. Self-compassion as a skill—personal attitudes, beliefs, and behaviors that SC could be developed and learned.	
2. Concordance between self-compassion and self-image—how men view the role of self-compassion within their view of themselves and failure/success	
• Masculinity gender norms	
• Personal views toward change and self	
3. The interplay of self-compassion and body image—how men view and feel about their bodies in relation to being self-compassionate	
• Body dissatisfaction/dysmorphia	Body satisfaction
• Body checking	
4. Self-Compassion in action—examples of how they treat themselves compassionately	
• Spirituality	Cognitive flexibility
• Self-care behaviors as a form of self-compassion Self-acceptance	Acceptance of circumstances
• Self-Acceptance	
5. Barriers to practicing self-compassion—impediments that make it more challenging for men to practice SC.	
• Weight cycling	Internalized weight stigma
• Restrict/rebound cycle	Perpetuating weight stigma (toward others)
• Experienced weight stigma	Toxic masculinity Upbringing
6. Facilitators to practicing self-compassion—things that support men’s practice or experiences with self-compassion	
• Upbringing	Age and Life Experience
7. Self-Compassion as a mask—signs of disordered eating, and compensatory behaviors referred to as self-compassion	
• Compensatory exercise as self-compassion	

### Self-compassion as a skill

Participants framed self-compassion (SC) not as a trait they inherently possessed but as a skill that could be learned and strengthened over time. Many described their current understanding of SC as a product of intentional effort, self-reflection, and lived experience. Some participants noted that this involved learning to acknowledge their imperfections without self-condemnation and instead using setbacks as opportunities for growth. As one participant described, SC meant “being fair with yourself,” and recognizing that mistakes are part of being human while engaging in self-reflection to avoid repeating them (see Table III). This framing emphasized the role of self-awareness and cognitive flexibility. Rather than catastrophizing failures or ruminating on perceived inadequacies, participants expressed efforts to reframe challenges with a more balanced inner dialogue. For example, men discussed the importance of not “beating yourself up,” being able to “look at the bright side,” and developing a “strong understanding of your own issues” as foundational to practicing SC.

**Table III.** Example quotes to illustrate relevant themes and sample subthemes.

Theme Subtheme	Quote
Self-compassion as a skill	
	“*I think [self-compassion] would be a learned skillset. Because I don’t know if you can necessarily just treat yourself that way [with self-compassion] without knowing how to.*”
	“*I would say self-compassion is probably more necessary in times when you're trying to lose weight. Because that's when you're really trying to do all the self-improvement, and you're really starting to take a deep look at yourself. And at that point is where it's probably when you need it the most. You need that hug on the inside.*”
Concordance between self-compassion and self-image	
• Masculinity gender norms	“*Maybe I’m seeing self-compassionate is accepting blame a little bit instead deflecting it. And that may just be the way that I’m wired, where there’s cause and effect and who’s the cause and what’s the effect. And if you’re the cause of the issue, then why be compassionate about it? Either you change it or you don’t.*”
• Views self as able to change	“*I think people put up their own barriers and then they start to believe them. And it’s very difficult to break out of those. And I’m as guilty as anybody else at saying, yeah, I’ll never do this. I won’t be able to do this. This is not what I’m good at. But I think if a situation came up where I needed to or wanted to get better at something, I have the tools within myself to do that.*”
The Interplay of self-compassion and body image	
• Body dissatisfaction/dysmorphia	“*I feel better because I have lost weight. I would say there was shame when I was heavier for sure.*”
• Body checking	“*I guess my biggest issue was the way I looked. I mean, I saw pictures of myself. I just didn't want to look at them just because I couldn't stand looking at them, I guess. I think that was the biggest reason why I started on [weight loss program], is just because of the way I looked.*”
• Body satisfaction	“*I feel better physically, a lot more energy. And then I look at myself differently. I'm more confident in what I do and say because I think people are looking at me for my ideas and my knowledge rather than looking at me and having thoughts about how big the dude is.*”
• Self-compassion in action	
Spirituality	“*When I start to feel inadequacies or there's a struggle with something, I don't beat myself up over it. I go to prayer and that's where I spend my time and I ask God, okay, I need your help here*.”
• Self-care behaviors as a form of sc	“*I make it a point to schedule time on my work calendar every day to break away for an hour, get out of the office, and take a walk. It's very important, and I put it on my schedule so that when people try to set up meetings or something like that, they see it as a, hey, I'm not available during this time.*”
• Cognitive flexibility	“*When my inner monologue kicks in, there is a lot of, hey, it's okay. It's not the end of the world. Failure is just a step to success.*”
• Acceptance of crcumstances	“*We learn to take care of ourselves. There's nothing you can do about it, just accept it and do what's expected of you and keep going.*”
• Self-acceptance	“*I think the best thing is to just accept yourself for what you are. I'm 62 so I'm really not going to be like, I kind of have been there and back already. I do things to make me happy. If it gives me satisfaction, then that's great. As you get older, it's just something you accept.*”
Barriers to practicing self-compassion	
• Weight cycling	“*The programs work.they allowed me to achieve a desired weight. On the other hand they didn't work, because I was unable to maintain the weight loss and.I basically blamed myself for.my inability to adhere to that program and to maintain the weight loss, but I also, I think. I also knew. That, if I was unable to maintain the weight loss, it was because the program was not really set up to do that.*”
• Restrict/rebound cycle	“*It's a lot of ebb and flow in I'm going to do this because I want to do this. I'm taking control. And then it's immediately followed by, oh my gosh, I've got to get control of my actions. I don't like where I'm headed. So it's this endless cycle of okay, starting tomorrow. We're going to get back on track. And then, you know what, I had a rough day. I'm having this apple fritter, dammit.*”
• Experienced weight stigma	“*So he [doctor] said, "You're still fine. You can still lose some more, but don't go anorexic or anything like that.*"
• Internalized weight stigma	“*I really think people judge a book by its cover and they don't take you seriously or you feel a little less valued. Some of that might be on how you see yourself and I don't know if that is a self-fulfilling prophecy, if you're feeling a little inadequate. So then you act maybe that way and then people start viewing you that way subconsciously.*"
• Perpetuating weight stigma (toward others)	“I think when you see somebody who's heavier, even as a person who was heavy, you just look at somebody and I guess you think of them as maybe lazy or don't take care of themselves.”
• Toxic masculinity	“.*our current society has been running off toxic masculinity for as long as, I mean, forever. Men aren't supposed to feel, and they're not supposed to have compassion. They're supposed to be tough and gritty and stuff like that. Especially in the older generations, I feel like there's a lot of toxic masculinity, a lot of men who have a hard time expressing themselves. And then that, I feel like self-compassion, is a high form of expressing yourself.*”
• Upbringing as a barrier	“*My upbringing happened in a culture, and I really think our culture. At least the culture that.has been present for most of my adult life.that culture is not very well aligned with this issue of self-compassion.*”
Facilitators to practicing self-compassion	
• Upbringing as a facilitator	“*.and my dad had a very typical dad approach to, ‘Well, I can't do this.’ No, it's not that you can't do it, it's just that you haven't practiced it enough. And then on the other side of the coin, my mom was very matronly in the, ‘You can do whatever you want. You just have to put your mind to it.’*”
• Age and life experience	“*I think I do think it's only it may be, in the last five or 10 years that I've started to become aware of the importance of self compassion.I think in my younger life. I was not very self-compassionate.*”
Self-compassion as a mask	
• Compensatory exercise as self-compassion	“*I think really needs to be focused on, not focused on, but just have an emphasis on, is really incorporating a physical component through exercise and activities that someone enjoys and is able to relax with. I think that incorporating the exercise will help with the self-compassion if someone were to not follow along perfectly. As I said, it's sort of a balancing, okay, you're going to have a little extra of this or that, or indulge in something. But if you balance it out with exercise and other things, then you can be compassionate to yourself, I guess.*”

Several participants tied the development of SC to their ability to make and sustain commitments, particularly in the context of health goals. One man reflected on whether to internalize failure (“I'm awful… might as well give up”) or to view emotional difficulty as part of change, granting himself the space to “be where I'm at” while still believing in his capacity to change. In this way, SC was not seen as an indulgence but a framework for persistence and resilience. This theme positions SC as an intentional mindset actively cultivated to support long-term behavior change.

### Concordance between self-compassion and self-image

This theme explores how men negotiated SC alongside deeply held values of discipline, responsibility, and personal control. While some participants embraced SC as a helpful and supportive mindset, others disagreed. Some men were concerned that SC might be misinterpreted as permissiveness or an excuse for failure.

Participants expressed tension between emotional self-kindness and the expectation of stoicism. Many men emphasized the importance of self-control, describing their efforts to “keep quiet and work through it” rather than dwell on setbacks. For some, this self-regulation was tied to masculine ideals of perseverance and restraint, shaping their perceptions of what “compassion” should look like in practice.

Some men questioned whether SC would undermine accountability, especially in the context of weight loss. One participant worried it might promote excessive self-forgiveness or complacency, framing this concern with language critical of those who “just complain” instead of taking action. For these men, personal responsibility remained paramount, and SC was only acceptable if it coexisted with action and results (i.e., weight loss).

In contrast, other participants offered a more flexible interpretation of failure, viewing it not as a weakness but as part of a longer growth process. These individuals described SC as accepting missteps without self-condemnation, to “keep going” rather than collapse under extreme perfectionism. This theme shows how men navigate cultural expectations of control, effort, and emotional expression in shaping their views of SC.

### The interplay of self-compassion and body image

Participants’ ability to practice SC was closely tied to their body image experiences, specifically body satisfaction, dissatisfaction, and checking behaviors. For many, weight loss brought a sense of pride and relief, helping them feel more comfortable in their bodies and more confident in their appearance. These physical and social shifts, fitting into new clothes and avoiding microaggressions or judgment, allowed some men to engage in more positive self-talk and project confidence onto others (see Table III).

However, the opposite was also expressed. When reflecting on periods of weight gain or living in a larger body, some men described discomfort, shame, and emotional distress. Several reported avoiding mirrors, photographs, or social situations altogether. This dissatisfaction was often cited as a catalyst for pursuing weight loss, but it also hindered their ability to relate to themselves with compassion in the present moment. In moments of shame or perceived failure, SC seemed inaccessible as it was overridden by self-criticism.

Body-checking emerged as a coping behavior some men used to regulate or monitor their distress. Participants described ritualistic practices like using clothing fit as a proxy for weight status or keeping “before” photos as cautionary reminders. While these strategies sometimes offered motivation, they also reinforced internalized weight stigma and fear of weight regain. This theme underscores how SC is not practiced in isolation from the body but is profoundly shaped by how men see and feel about themselves physically. For some, improvements in body image created the space for SC to flourish. For others, lingering dissatisfaction served as a barrier to self-kindness.

### Self-compassion in action

Participants identified specific ways they enacted SC in everyday life, such as reframing personal setbacks, reducing self-criticism, and redirecting energy into meaningful activities rather than dwelling on perceived failures. Rather than being consumed by guilt or negative self-talk, men described efforts to acknowledge imperfection without losing momentum. Some participants described practicing SC through a gentle internal voice, such as reminding themselves that it was acceptable to be a “work in progress.” For example, one man noted that while he still held himself to high standards, he no longer believed it was necessary to be excessively hard on himself. This balanced perspective allowed room for effort and acceptance to coexist (see Table III).

Other participants shared examples of shifting focus from weight loss to broader self-development. For one man who no longer saw weight change as feasible, SC meant expanding the definition of progress: nurturing other areas of life and investing in emotional and psychological well-being. These actions reflect a reorientation of self-care. Instead of SC being contingent on physical transformation, it became part of an ongoing process of self-respect. This theme illustrates that SC was not simply conceptual for participants; it was lived through flexible thinking, emotional regulation, and daily choices to engage kindly with oneself.

### Barriers to self-compassion

Participants described a range of barriers that made practicing SC difficult, especially in moments of perceived failure, weight regain, or emotional distress. Compared to facilitators, barriers were more frequently shared, suggesting that SC remained an unfamiliar or challenging concept for many men when they were not meeting weight loss goals.

A recurring theme was the restrict-rebound cycle standard in weight-normative contexts. Many participants described internal struggles with deprivation, followed by loss of control, self-criticism, and resignation. In these moments, SC was not used; instead, participants often defaulted to self-blame and reactive behaviors. Several men linked these cycles to yo-yo dieting and weight cycling, noting that shame and frustration often led them to “give up” rather than respond with self-kindness (see Table III).

Barriers also stemmed from internalized weight stigma. Participants spoke openly about feeling judged in public spaces, even when eating “healthy” foods. They believed their appearance made them fundamentally less worthy or less valued. Healthcare providers, workplaces, and social environments often reinforced these beliefs. As a result, many participants adopted self-deprecating narratives, which undermined their ability to respond to setbacks with compassion.

Several participants also reflected on how timing and readiness influenced their openness to SC. Early in pursuing weight loss, they were more likely to reject SC as indulgent or counterproductive and instead noted preferring structured plans and accountability over emotional support. One participant recalled that had SC has been introduced at the start of his weight loss program; he would have dismissed it as justification for making poor choices.

Finally, upbringing and cultural norms were cited as barriers. Men raised in rigid or emotionally restrained environments (such as those influenced by military culture or traditional masculine ideals) described SC as foreign. Compassion was not modeled or encouraged for them, and emotional regulation was framed as stoicism or self-discipline, not kindness. These barriers highlight the complexity of introducing SC into male weight-loss contexts. Without addressing underlying stigma, unlearning punitive thinking, and considering timing and context, efforts to cultivate SC may be met with resistance or misinterpreted as weakness.

### Facilitators to practicing self-compassion

Several participants identified key factors that helped them cultivate a more compassionate relationship with themselves. These facilitators included early prosocial messaging from caregivers and the perspective that came with aging and life experience. Some men described being raised in environments where kindness, emotional resilience, and care for others were emphasized. For one participant, messages from both parents encouraged self-reliance and patience. These values shaped his ability to treat himself with the same empathy he extended to others and laid a foundation that later supported SC in adulthood.

Aging also appeared to shift how men responded to challenges. With time and experience, participants described becoming less rigid and more accepting of imperfection. They acknowledged that difficult emotions and failure were inevitable and that emotional self-harshness was neither valuable nor sustainable. In contrast to earlier phases of life where self-criticism was more dominant, older participants reported greater emotional flexibility and a stronger internal belief that “it’ll be okay” (see Table III). This theme suggests that SC may be more easily adopted when men have internalized compassion values from trusted role models and have lived through enough setbacks to recognize the limits of self-punishment.

### Self-compassion as a mask

Some participants described behaviors they believed reflected SC, such as overindulging in food or compensating for eating food with exercise that appeared to reflect disordered or avoidant patterns. These narratives suggested that SC was sometimes co-opted to justify emotional eating or punitive exercise cycles rather than to support well-being. In particular, men spoke about eating in response to cravings and later struggling with guilt, often rationalizing indulgent behaviors as deserved or necessary. In hindsight, these behaviors were often recognized as unhelpful but were still framed as self-care in the moment. Others described using exercise to compensate for caloric intake, equating “balance” with burning off indulgences rather than treating the body with compassion.

These accounts raise concern, as participants did not typically recognize these patterns as disordered. Participants viewed these acts as self-kindness even when the behaviors aligned with definitions of irregular or compulsive eating and exercise. This lack of recognition is significant given that dieting and weight-focused behaviors are proximal risk factors for disordered eating (DerMarderosian & Hall, 2011; Tang et al., 2023). The framing of these compensatory patterns as acts of SC suggests a blurring of boundaries between self-kindness and self-discipline, where control and punishment may masquerade as compassion. Without clear, emotionally grounded models of SC, men may reinterpret harmful behaviors as helpful coping strategies. Future work should explore how SC is communicated and modeled in weight-normative contexts to ensure it supports mental and physical health.

## Discussion

SC is an affect-regulation strategy that can improve nutrition behaviors, overeating, and body dissatisfaction; however, most studies incorporate only female samples (Rahimi-Ardabili et al., 2017). Qualitative research examining SC in male athletes shows that young men who participate in sports are open to embracing SC to enhance their athletic performance (Reis et al., 2022) and that male athletes use SC to cope with sports and life-related challenges (Tremblay et al., 2023). Prior research examining SC in men has shown that traditional masculinity norms are associated with reduced help-seeking attitudes in intercollegiate male athletes (Wasylkiw & Clairo, 2018). Of the few studies that examine SC in men, the majority examine male athletes who compete at the collegiate level (Reis et al., 2019; Reis et al., 2022; Tremblay et al., 2023). The present study is aligned with recommendations for advancing applications and research on SC in men since we exclusively recruited this study sample outside of the sports context (Reis et al., 2019). Qualitative research can inform and guide clinical practice, especially when examining the perspectives and experiences of groups not well-represented in the literature (Hammarberg et al., 2016). This study provides insight into unique barriers and facilitators that adult men pursuing weight loss experience when practicing SC.

Through reflexive engagement with the data, we developed seven key themes across 11 in-depth interviews: 1) SC as a skill, 2) Concordance between SC and Self-Image, 3) The Interplay of SC and Body Image, 4) SC in Action, 5) Barriers to Practicing SC, 6) Facilitators to Practicing SC, and 7) SC as a Mask. Men described SC as conditionally accessible, often dependent on achieving weight loss, reflecting the entanglement of self-worth with body size.

Several men reported restrict/rebound cycles and weight cycling and acknowledged that shame was a common experience when they were navigating the world in a larger body. This finding is aligned with research that shows perceived stigma and self-stigma (i.e., internalized weight stigma) are positively associated with psychological distress (Alimoradi et al., 2020). These results are particularly concerning for people pursuing weight loss, as weight regain after such efforts are common (Kraschnewski et al., 2010; MacLean et al., 2011; Wing & Phelan, 2005). The psychological effects of yo-yo dieting could lead men toward experiencing greater shame and guilt and, subsequently, lowered SC. Body image was a crucial factor that men discussed, such that body dissatisfaction, body dysmorphia, body checking behaviors, and body satisfaction all influenced whether men felt self-compassionate. The relationship between body image and SC expressed in this study reflects other research on body image in men, showing that lower body satisfaction is linked to increased self-criticism, which can lower SC (McFarland & Kaminski, 2009). Further, exposure to idealized body images can worsen body dissatisfaction and reduce SC (Arbour & Ginis, 2006; Turk et al., 2021).

Men noted significantly more barriers than facilitators when it came to SC. These barriers primarily focused on weight-related experiences, including weight stigma (perceived and internalized) and weight cycling. These barriers are different from the internal and external barriers that women seeking weight loss noted in Jeziorek and Riazi (2022). When examining narratives of SC in women seeking weight loss, Jeziorek and Riazi (2022) noted that women experienced the following barriers: they struggled to put their needs first over the needs of others, had extremely high standards of self, and how early life experiences served as a hindrance to practicing SC (Jeziorek & Riazi, 2022). These results differ slightly from the findings presented herein and could speak to the importance of tailoring SC programming to different populations. These findings suggest that interventions incorporating SC for men must first address underlying stigma, masculine norms, and distorted body image beliefs. Without these foundations, SC risks being co-opted as justification for disordered behaviors rather than a tool for healing. Extant research overwhelmingly supports that internalized weight stigma and perceived weight stigma from others are negatively associated with SC; however, this research mainly examines female samples (Fekete et al., 2021; Forbes & Donovan, 2019). Future work should include studying associations between SC and internalized and perceived weight stigma in men.

Finally, several participants described SC in ways that could be interpreted as concealing disordered behaviors. The relationship between dietary restraint and eating disorder development is complex, and it is believed that dieting may trigger disordered eating and eating disorders in people who are already vulnerable or susceptible (Stewart et al., 2022). The complexities involved in dietary restraint leading to disordered eating behaviors may explain why some participants expressed SC as a form of dietary restriction and compulsive exercise while others did not. It is possible that men, who already face more difficulties in recognizing and seeking help for eating disorders compared to women (Grillot & Keel, 2018), may not identify certain behaviors as motivated by body dissatisfaction or body shame as opposed to self-kindness. Regardless, SC as a guise for compensatory restriction and exercise is an area that warrants further study.

### Strengths and limitations

The present findings should be considered in the context of study limitations. As is common in qualitative research, this study included a small, purposive sample for rich, in-depth analysis. While the findings are not intended to be generalizable, they offer insight into key patterns of meaning that may inform future research and practice. Another limitation involves the lack of a comparison group. This study focuses solely on participants' experiences without comparing them to adult men not enrolled in a weight loss program or not actively seeking weight loss. It would be interesting for future work to incorporate a comparison group of adult men in larger bodies who are not actively pursuing weight change to determine if perceptions and experiences with SC are different across those groups. Further, we interviewed a homogenous sample of all non-Hispanic White men, and this lack of diversity limits the transferability of study findings to other ethnic groups and individuals not enrolled in weight loss programs. That said, we aimed to deepen our understanding of how men seeking weight loss interpret and engage with self-compassion to inform more effective integration of SC practices in future interventions targeting this population.

There are several notable study strengths. Reflexive thematic analysis allowed for an in-depth and flexible exploration of participants' experiences, supporting the identification of rich, nuanced patterns of meaning across narratives. The interview process was also thorough and incorporated rapport-building and member checks, improving the validity of the study findings. Though the intention of this study is not to generalize, our sample encompassed a wide age range (26 to 77 years), ensuring comprehensive representation across different generational cohorts. Furthermore, participants were provided an operational definition of SC from the literature to ensure a common understanding of the subject before asking questions related to their body image, behaviors and habits, and perceptions of barriers and facilitators to the practice of SC. Participants were interviewed through video conference, which provided flexibility and could have potentially improved rapport since they were interviewed in their chosen environment. The study followed what Cofie and colleagues (2022) recommend for generating reliable study findings across individual team members in qualitative research. Finally, the novelty of this work and its significance in health coaching cannot be overstated. Previous SC studies have predominantly focused on female samples (Ferrari et al., 2019), leaving a notable gap that this research aims to fill.

### Clinical impact and future directions

Health coaching has emerged as a practical approach to addressing chronic conditions and improving health and well-being among individuals (Sforzo et al., 2020; Thom et al., 2015). Still, much of the research on health coaching focuses on weight-normative programs encouraging weight loss despite growing concerns that the underlying assumptions behind weight loss are faulty and problematic (Hunger et al., 2020). Furthermore, studies show that men who experience disordered eating are more likely to seek weight loss treatments than treatment for eating disorders (Mars et al., 2019; Westerberg & Waitz, 2013). The present study examines how SC is viewed and perceived by men enrolled in a weight loss program that incorporates health coaching. An improved understanding of how men experience SC could be applied to weight-normative health coaching programs to help men cope with body image issues and weight bias internalization. The integration of SC with men could be beneficial for health coaches who practice from a weight-inclusive approach and may lead to improved body acceptance, body appreciation, and eating behaviors (Burychka et al., 2021; Swami et al., 2019; Turk & Waller, 2020). Our findings are aligned with the 5 C framework, a guiding framework that details five components of program development and design aimed at transforming approaches to men’s health (Galdas et al., 2023). In light of the current study findings and the 5 C framework, future research should consider utilizing a community-based participatory research (CBPR) approach to build SC interventions for men in health coaching programs. CBPR has been shown to enhance the efficacy of interventions but has been relatively underutilized in psychology (Rodriguez-Espinosa & Verney, 2021; Wallerstein & Duran, 2010). An intervention utilizing SC with men who are seeking health coaching services could address topics such as body image, disordered eating, weight-related distress, loss of control of eating, and weight bias internalization.

## Conclusions

This study is the first to qualitatively explore experiences with SC in adult men pursuing weight loss treatment with a history of dieting. Our findings reflect the researchers’ interpretive engagement with participants’ meaning-making rather than a neutral experience description. There were seven noteworthy themes that need to be considered for future interventional work in the context of weight loss, including barriers to practicing SC and SC as a mask for disordered eating and exercise behavior. These findings suggest that interventions must go beyond generic SC education to actively address stigma, challenge harmful masculine norms, and create space for more adaptive body image narratives. For example, future programs or interventions could account for masculine norms by framing SC in terms of strength and resilience rather than softness to increase receptivity. Our work also points to the necessity of addressing weight cycling and internalized weight stigma as these were noteworthy barriers. Recent developments to address internalized weight stigma include a cognitive-behavioral intervention to be used in weight management settings (Pearl et al., 2022). Still, others posit that the only way to truly reduce internalized weight stigma and reduce eating disorder risk is to stop promoting weight loss (Steinberg & Bohon, 2022). Future intervention work could consider embracing a CBPR approach. By actively involving adult men who have undergone dieting experiences, this collaborative framework could serve as a compass guiding the design and implementation of interventions that resonate with the target demographic's realities and needs. These insights reflect the perspectives of men engaged in commercial weight loss treatment with health coaching and may not extend to broader populations. The present study lays the groundwork for future observational and experimental research on SC in adult men and underscores the need for context-specific adaptations to SC practice for men seeking weight loss, while recognizing that further research is needed to explore applicability in other settings.

## Supplementary Material

Supplementary Material Communication with Adult Men with Active Profile by Sanford Health Memberships

Supplementary Material Exploring Self-Compassion Among Men Seeking Weight Loss: A Thematic Analysis

## Data Availability

The data supporting this study's findings are not publicly available due to the sensitive and identifiable nature of the qualitative transcripts. However, de-identified excerpts may be available from the corresponding author upon reasonable request and with appropriate ethical approval.
