# Supplementary material for: Exploring self-compassion among men seeking weight loss: a thematic analysis
Source: Int J Qual Stud Health Well-being. 2025 Nov 8;20(1):2577285. doi: 10.1080/17482631.2025.2577285 (PMC12599162; doi:10.1080/17482631.2025.2577285)
Supplement: Supplementary Material — Communication with Adult Men with Active Profile by Sanford Health Memberships [file ZQHW_A_2577285_SM8989.docx]

**Communication with Adult Men with Active Profile by Sanford Health Memberships**

Interviewer:

*Recording set to start as meeting begins*

Thank you so much for taking the survey and for being willing to participate in this follow-up interview. We are working at Profile to understand unique programs we can offer to members to support their health goals and improve overall wellness. As a doctoral student at NAU I am working with the research and innovation team at Profile to do research to advance this work. So, this is an interview, but it is also a chance to find out more about your experiences and discuss how Profile can best support other members with similar experiences in the future.

The first thing we need to do is discuss the consent form for the interview. [Give them the consent form.] Would you prefer to read through this yourself or would you like us to talk through it together?

Do you have any questions? If this sounds OK to you, please sign the form and indicate whether it is OK for me to audio record [and whether you would prefer to be anonymous or have your real name used].

*If participant declines recording, thank them for their time and let them know that the interview cannot continue*

**First, I’d like to learn a little bit about you and your experiences.**

1. You’ve been a Profile member for X months/years? How has your experience been with Profile so far?

2. What prompted you to join Profile by Sanford?

3. What has been the best part of the Profile by Sanford program based on your experience so far?

**Now I’d like to learn more about your experiences at your weight previously and currently.**
4. When you think back to your highest weight, what was your experience like? Were you treated differently by other people or in certain places/environments based on your weight?

4a) When you think about being at your highest weight, how were you treated differently then you are at your current weight?

5. What is your experience of being your current weight?
6. How do you feel about your self-image? Do you think your weight has any impact on your self-image?

**I’d like to ask you some questions specifically about your experiences with self-compassion.**

7. How do you define self-compassion?

*Share our definition of self-compassion*

Self-compassion is acknowledging that suffering, failure, and inadequacies are part of the human condition, and that all people—yourself included—are worthy of compassion.

Now, using our definition of self-compassion:

8. Is self-compassion something you think about day to day or in relation to yourself?

Examples could be acknowledging you’re going through something difficult while it is happening instead of trying to ignore it, or mentally saying something kind to yourself when you’re struggling.

9a) Tell me more…

9. What were your experiences of self-compassion while you were growing up?

10a) How about compassion toward others/from others?

10. What have been your experiences of self-compassion more recently?

11a) How about compassion toward others/from others?

11. Do you perceive yourself to experience any barriers to self-compassion? What do you

think may be most contributing to these?

11a) How do you feel about these barriers?

11b) Do you think barriers to self-compassion may be related to your wellbeing?

11c) What would it mean to have fewer barriers to self-compassion in the future?

12. How do you experience self-compassion while trying to lose weight? Is it similar to times you’re not trying to lose weight?

Do you think SC is an important element in a program like profile? Why?

**Finally, I’d like to talk about your experiences with dieting or intentional weight loss.**

13. What is your experience of deciding to go on another diet after you completed one?

13a) How did you talk to yourself/treat yourself during that process?

13b) How are you taking care of yourself on a day to day?
